# Supercooling as a Viable Non-Freezing Cell Preservation Method of Rat Hepatocytes

**DOI:** 10.1371/journal.pone.0069334

**Published:** 2013-07-16

**Authors:** O. Berk Usta, Yeonhee Kim, Sinan Ozer, Bote G. Bruinsma, Jungwoo Lee, Esin Demir, Tim A. Berendsen, Catheleyne F. Puts, Maria-Louisa Izamis, Korkut Uygun, Basak E. Uygun, Martin L. Yarmush

**Affiliations:** Center for Engineering in Medicine at Massachusetts General Hospital, Harvard Medical School and Shriners Hospital for Children, Boston, Massachusetts, United States of America; The Ohio State University, United States of America

## Abstract

Supercooling preservation holds the potential to drastically extend the preservation time of organs, tissues and engineered tissue products, and fragile cell types that do not lend themselves well to cryopreservation or vitrification. Here, we investigate the effects of supercooling preservation (SCP at -4^o^C) on primary rat hepatocytes stored in cryovials and compare its success (high viability and good functional characteristics) to that of static cold storage (CS at +4^o^C) and cryopreservation. We consider two prominent preservation solutions a) Hypothermosol (HTS-FRS) and b) University of Wisconsin solution (UW) and a range of preservation temperatures (-4 to -10 ^o^C). We find that there exists an optimum temperature (-4^o^C) for SCP of rat hepatocytes which yields the highest viability; at this temperature HTS-FRS significantly outperforms UW solution in terms of viability and functional characteristics (secretions and enzymatic activity in suspension and plate culture). With the HTS-FRS solution we show that the cells can be stored for up to a week with high viability (~56%); moreover we also show that the preservation can be performed in large batches (50 million cells) with equal or better viability and no loss of functionality as compared to smaller batches (1.5 million cells) performed in cryovials.

## Introduction

There are currently three outstanding questions in the field of preservation 1) How can we preserve whole organs to improve quality and logistics issues in transplantation? 2) Can we devise an effective preservation scheme to ease the dissemination of the growing number of engineered tissue products? 3) Can we preserve primary cells with metabolic and enzymatic activity similar to freshly isolated cells in a robust and cheap manner? And finally one can ask the question if we can answer these three questions with the aid of a unified method.

Here, in an effort to build a preservation method towards answering these questions, we use a strategy inspired by freeze avoiding species such as fish, amphibians and insects [1–4]. Such species can supercool their contents to weather the long and cold winters, with temperatures that can go as low as -45^o^C [5], and still survive. The earliest works on supercooling preservation (SCP) date back to the 1960s with storage of bacterial and yeast cells [6,7] followed intermittently with other cell types such as, peripheral blood stem cells [8], turkey spermatozoa [9,10], cells of various rat organs [11–15]. More recent studies also include short term organ storage examples on heart [16,17], liver [16,18–21], lung [22], and kidney [16]. The temperature range that has been studied in these cell and organ studies goes from slightly below 0 ^o^C for organs [16–23], to -5^o^C for mammalian cells [8–15], and all the way down to -30 to -40 ^o^C for bacterial and yeast cells [6,7]. While some of the earlier work has produced some controversial and conflicting results about the effects of SCP on viability and function at extremely low temperatures (≤-10^o^C), more recent research has focused on around the -5 to 0 ^o^C window with more coherent results in terms of viability and function. Nevertheless, most of these works are limited in preservation period and lack a complete panel of functional assessments;a significant number of them are limited to resilient cell types and/or cells which proliferate in vitro therefore high viability is not critical. A long-term solution for primary cells and fragile cells such as hepatocytes remains missing.

In this work we aim to establish SCP as a feasible approach for extended preservation of hepatocytes. Since metabolism is a sum of temperature dependent reactions [2], we expect that lowering the temperature beyond static cold storage temperature (+4^o^C) should result in a favorable slowing down therefore a lower need for oxygen and energy consumption. We hypothesize that there exists an optimum supercooled temperature at which metabolic processes are slow enough to extend preservation times dramatically beyond current hypothermic approaches (about 24 hours for suspended hepatocytes on ice) while avoiding injuries caused by strategies that involve phase transitions such as cryopreservation and vitrification. With this in mind, in SCP, we aim to combine the best features of static cold storage (robustness and easy handling) and cryopreservation (long preservation times) while avoiding their shortcomings and complications. To this end our first aim was to lower the preservation temperature below +4^o^C while still avoiding phase changes and crystal formation. A secondary aim was to avoid costly equipment that is required for precise handling of cryopreservation procedures and use cheap, portable, temperature controlled chillers/fridges that are already common place and used for storage and transport of blood.

In what follows, we present a comprehensive study comparing different preservation solutions and a range of temperatures for small and large batches of cells. We compare our results with conventional preservation methods where possible. In section 1 a) we first present the viability, energy storage and short term suspension functionality (albumin and urea secretions and EROD activity) of cells preserved (up to a week) in small cryovials holding 1.5x10^6^ cells; here we will discuss the existence of an optimum temperature (-4^o^C) and an optimum preservation solution (HTS-FRS) based on these results; we also present further evidence for the success of this combination using long term plate culture studies of the preserved cells in section 1 b). In section 1c) we show that that the optimum subzero non-freezing storage protocol can also be scaled up and conducted in batches of 50x10^6^ cells in regular centrifuge tubes with better viability and equal/better functionality in suspension and plate cultures. Finally, we present a scanning electron microscopy imaging study of the preserved cells to assess the extent of external cell membrane damage among different preservation schemes in section 1 d). Finally how these studies at the cellular level may be translated into tissue and organ preservation is discussed.

## Results

### A. Suspension Culture Viability and Functionality of Preserved Cells

We investigated the preservation of rat hepatocytes in two commercially available preservation solutions that aim to preserve intracellular balance; these are a) HTS-FRS (Biolife Solutions Inc., referred to as HTS herein), and b) University of Wisconsin (UW) solution, the gold standard in preserving livers and other organs. All experiments were conducted in 1.5 ml Nalgene® cryovials except where noted.

**Figure 1:**
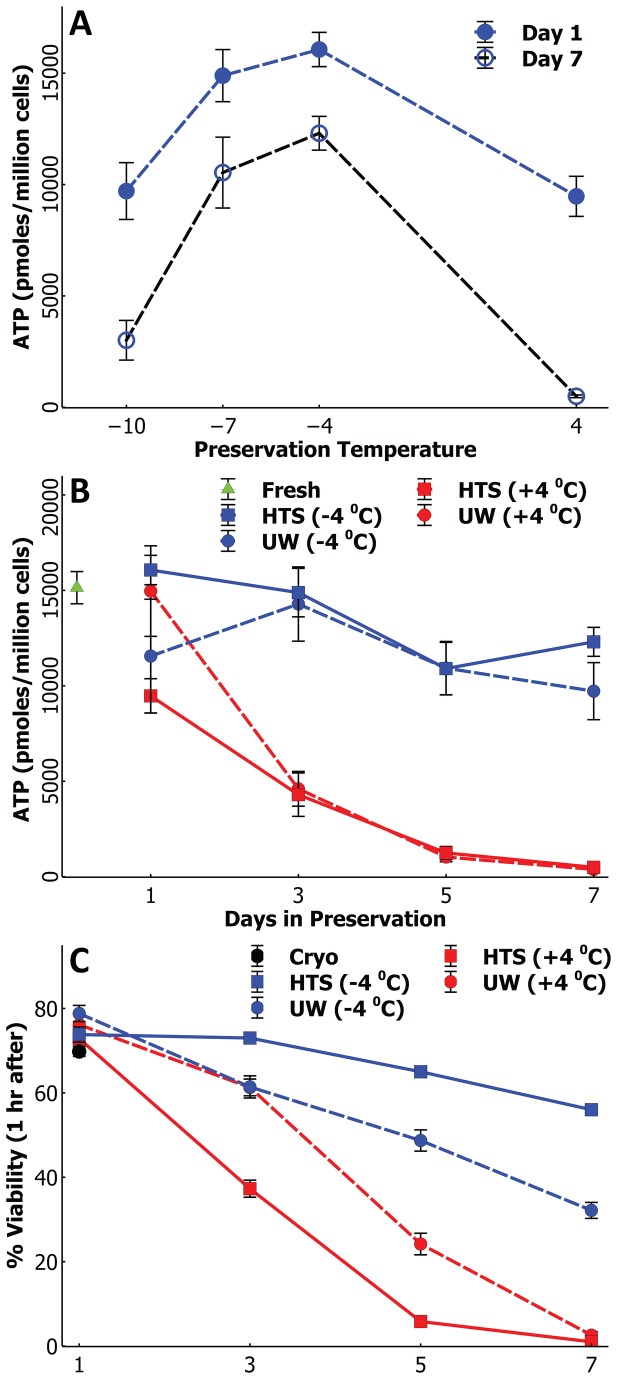
ATP depletion and viability comparison of supercooled rat hepatocytes with other preservation modalities. A) Comparison of ATP retention of cells at different temperatures in HTS solution; cells preserved at -4 and -7 ^o^C retain statistically similar amounts of ATP (p>0.05) while ATP retention at both temperatures are significantly higher compare to -10 and +4^o^C on both days B) Comparison of ATP retention for cells preserved in HTS and UW solutions at +4 and -4 ^o^C. ATP retention among the two solutions are statistically similar (p>>0.05) at either -4 or 4 ^o^C; however, there is a a statistically significant (p<0.05) difference in ATP retention between -4 and at 4^0^C at and beyond 3 days of preservation. C) Cell viability after 1 hour of suspension rewarming monitored up to 7 days of preservation; any two values that do not have overlapping error bars on this graph is significantly different from each other (p<0.05).

In order to find the optimum temperature for SCP we assessed the ATP retention of cells stored for 1 day and 7 days at four different temperatures in a range of -10 to +4 ^o^C (Figure 1 A); ATP was measured only in vials that showed no crystallization. We find that ATP retention is markedly different at different temperatures with one emerging trait: the overall retention is the highest in cells preserved at -4^o^C and -7^o^C on both days. While one would expect ATP retention to increase as the temperature decreases based on the Van’t Hoff principle, the sudden decline of ATP at -10^o^C is likely a result cellular and organelle membrane damage, and other intra-cellular injuries giving rise to cell apoptosis. Nevertheless, these signify that cells stored -4^o^C and -7^o^C are likely to have the highest viability and retain the highest function. Another important issue in SCP is the probabilistic occurrence of freezing or slush formation during storage especially at the lower temperatures that we studied (-10^o^C and -7^o^C). At -10^o^C, the percentage of solutions that survive (i.e. no freezing) supercooling is close to 50% after 24 hours and is down to 15% after 7 days; at -7^o^C the survival rates are about 80% and 50% for 1 and 7 days of storage. At -4^o^C the survival rate is close to 100% after 24 hrs and down to 95% after a week. Together with the high ATP retention, this high supercooling success (low probability of freezing) over a week justifies the use of -4^o^C as the optimum temperature for our SCP experiments without use of additional antifreeze components such as anti-freeze proteins.

We then moved on to elucidate the differences between UW and HTS solutions for SCP of rat hepatocytes at -4^o^C compared to the gold standard of CS at +4^o^C. In Figure 1 B we present the ATP retention of cells preserved for 1, 3, 5, 7 days in UW or HTS at -4^o^C and +4^o^C. We show that the choice of preservation solutions does not significantly affect the ATP retention; cells stored in either solution at -4^o^C show a slow decline while those stored at +4^o^C show a very rapid depletion of ATP reserves to almost zero by the end of one week. Fitting an exponential decay model, of the form e^-at^, where a is the time constant and t is the time in days, to the curves in Figure 1 B) reveals that the depletion is about 10 times slower at -4^o^C compared to +4^o^C.

ATP retention is considered a good early indicator of cell viability and function, thus following the above ATP experiments we set out to assess the detailed fate of preserved cells using both viability and functional assessments; from here on we used all the four non-freezing preservation groups – i.e. cells preserved a) in HTS at -4^o^C b) in UW at -4^o^C c) in HTS at +4^o^C d) in UW at +4^o^C - alongside a cryopreserved group; In Figure 1 C we compare the viability, via Trypan blue exclusion, of all preserved groups following a 1 hr suspension rewarming at 37^o^C, which is a common early test for cell viability. We show that, after one day of preservation all non-frozen groups show a similarly high 1-hr viability close to 80% (Figure 1 C), higher than that of cryopreserved group (~70%). By 3 days of preservation the cells preserved in HTS at -4^o^C perform better than all other groups; this group continues its high 1-hr viability even after a week of preservation (~56%) – this viability is significantly higher than those measured both in UW SCP group and the CS (+4^o^C) groups. Cells preserved at + 4^o^C in either solution show a rapid decline in 1-hr viability and drop to almost zero at the end of a week; however, cells preserved in UW at +4^o^C show significantly better viabilities on the third and fifth days compared to the HTS solution. It’s notable that HTS performs best at -4^o^C while UW performs better at +4^o^C.

**Figure 2:**
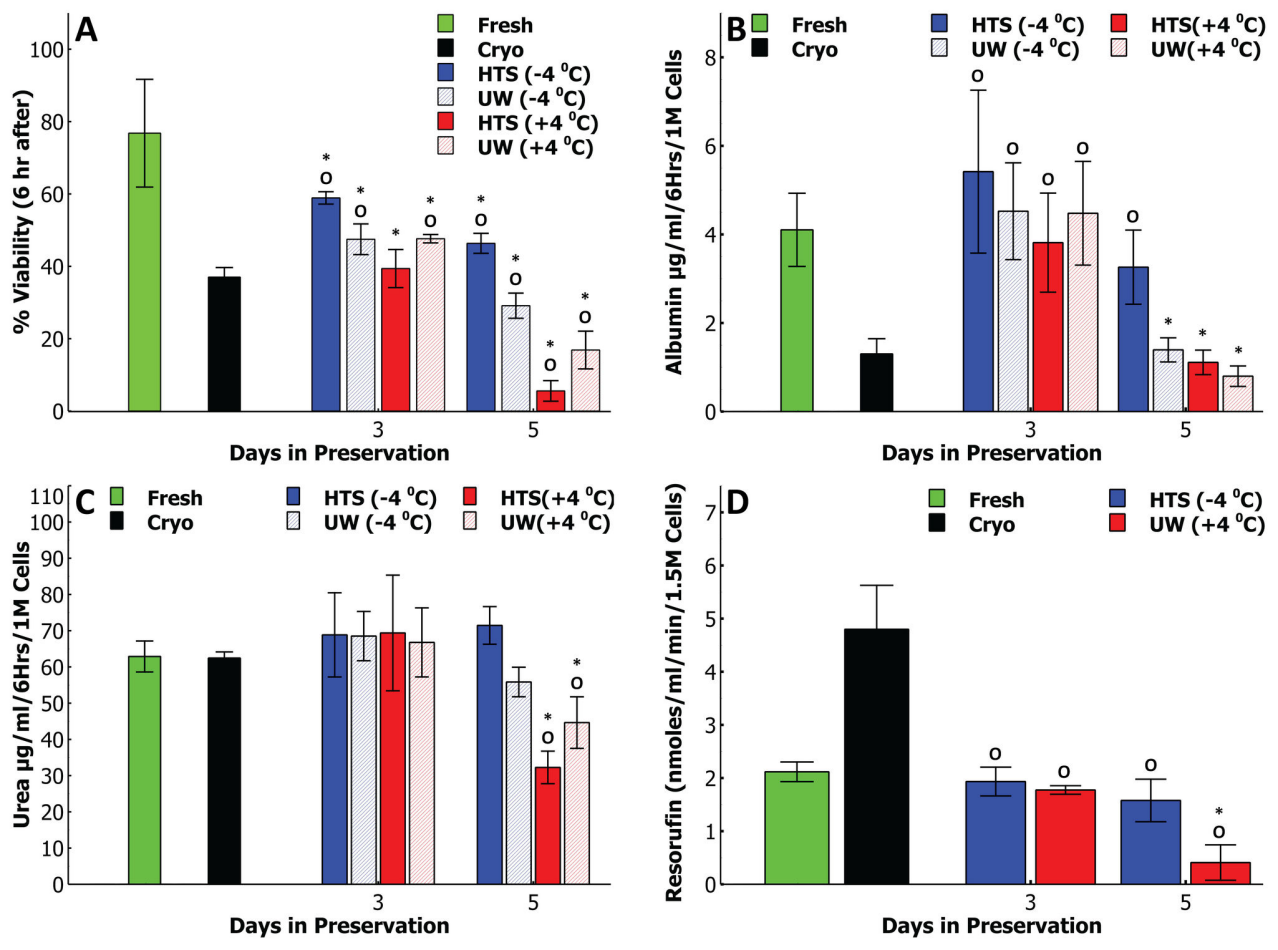
Suspension function comparison of the supercooledrat hepatocytes with other preservation modalities and fresh cells. A * and ^O^ means statistically different (p<0.05) than fresh and cryopreserved cells respectively. A) Cell viability after 6 hours of suspension culture; HTS at -4^o^C group has significantly higher (p<0.05) viability than all other groups (except fresh cells) on both days. B) Albumin production by cells during 6 hours of cell suspension solution. All groups except cryopreserved cells show similar albumin secretion on day 3 while HTS at -4^o^C group produces significantly more (p<0.05) albumin than all other preserved groups on day 5 which is statistically similar (p>0.05) to the fresh cells. C) Urea production by cells during 6 hours of cell suspension. The urea production by all preserved groups except cryopreserved cells is statistically similar (p>>0.05) to fresh cells on day 3; by day 5 of preservation the two groups preserved at -4^o^C produce statistically similar levels (p>0.05) of urea to that of fresh cells which is significantly different (p<0.05) than cells preserved at +4^o^C. D) Resorufin production by preserved cells using ethoxy coumarin as the substrate (EROD analysis). Enzymatic activity among two non-frozen groups and fresh cells is similar after 3 days of preservation. After 5 days of preservation the HTS at -4^o^C group is statistically similar (p>0.05) to fresh cells and significantly better (p<0.05) than UW at +4^o^C group. Cryopreserved cells show significantly higher levels of enzymatic activity compared to all other groups.

Next we have assessed viability and functionality of cells preserved for 3 and 5 days (Figure 2) following a 6 hour suspension culture at 37^o^C; a suitable scenario for short term use cases such as ADME-Tox tests. Cells preserved in HTS at -4^o^C still show the highest viability even after 6 hrs, for both 3 and 5 day preserved cells, compared to all other groups. Remarkably, cryopreserved cells show a drastic decline from the first to the sixth hour, down to 37%, indicating they are less than ideal for 6hr suspension testing. Among SCP and CS groups, the decline from 1 hr to 6 hr viability compared to 1 hr viability is ~ 20% on third day and increases to a 30-40% drop on fifth day.

Figures 2 B and 2 C confirm that cells preserved in HTS at -4^o^C outperform all other groups including cryopreserved cells, especially for five day storage, in terms of functional secretions and excretions. Albumin secretion and urea excretion of cells preserved for 3 days (Figure 2 B & C) are statistically similar. Among cells preserved for 5 days in HTS at -4^o^C group stands out with significantly higher secretions/excretions (p<<0.05 in all pairwise comparisons) especially for albumin. We see that cells preserved in HTS at -4^o^C show not only the highest secretion activity but also a very similar one (p>>0.05) to that of freshly isolated hepatocytes even after the fifth day of preservation.

An important test for the functionality of the preserved cells is to assess their drug metabolizing enzyme activity; the retention of enzymatic activity is not only important for eventual cell and organ transplantation purposes but also for use in cell based drug metabolism and toxicity studies. To this end we have employed a suspension EROD (CYP1A1) analysis; here we have reduced the experimental groups to include only the cells preserved in UW at +4^o^C and HTS at -4^o^C along with fresh control group and the cryopreserved cells. Inline with our previous suspension analyses, we observe that, among the non-frozen preserved groups, cells preserved at -4^o^C show a higher enzymatic activity than the +4^o^C group (p<<0.05) especially on the fifth day and one that is statistically same as fresh cells (p>>0.05). An unexpected result during these experiments were the relatively high activity (more than twice that of the fresh group) shown by the cryopreserved cells; although we hypothesize that this might be a result of cell lysis and an subsequent release of CYP450 enyzmes, this result does require further investigation which is beyond the scope of this paper.

### B. Long Term Plate Culture of Preserved Cells

An important assessment of cell fate after preservation is attachment to an extracellular matrix and proper functioning thereafter; therefore, we cultured preserved cells from each group in a 24-well collagen sandwich format for up to a week. During this period we assayed samples at 1, 3, 5 and 7 days of culture. The cultures were terminated if microscopic assessment indicated low confluence and/or low viability; none of the 7 day preserved groups were cultured beyond 24 hours as they have all shown low attachment and loss of differentiated morphology.

**Figure 3:**
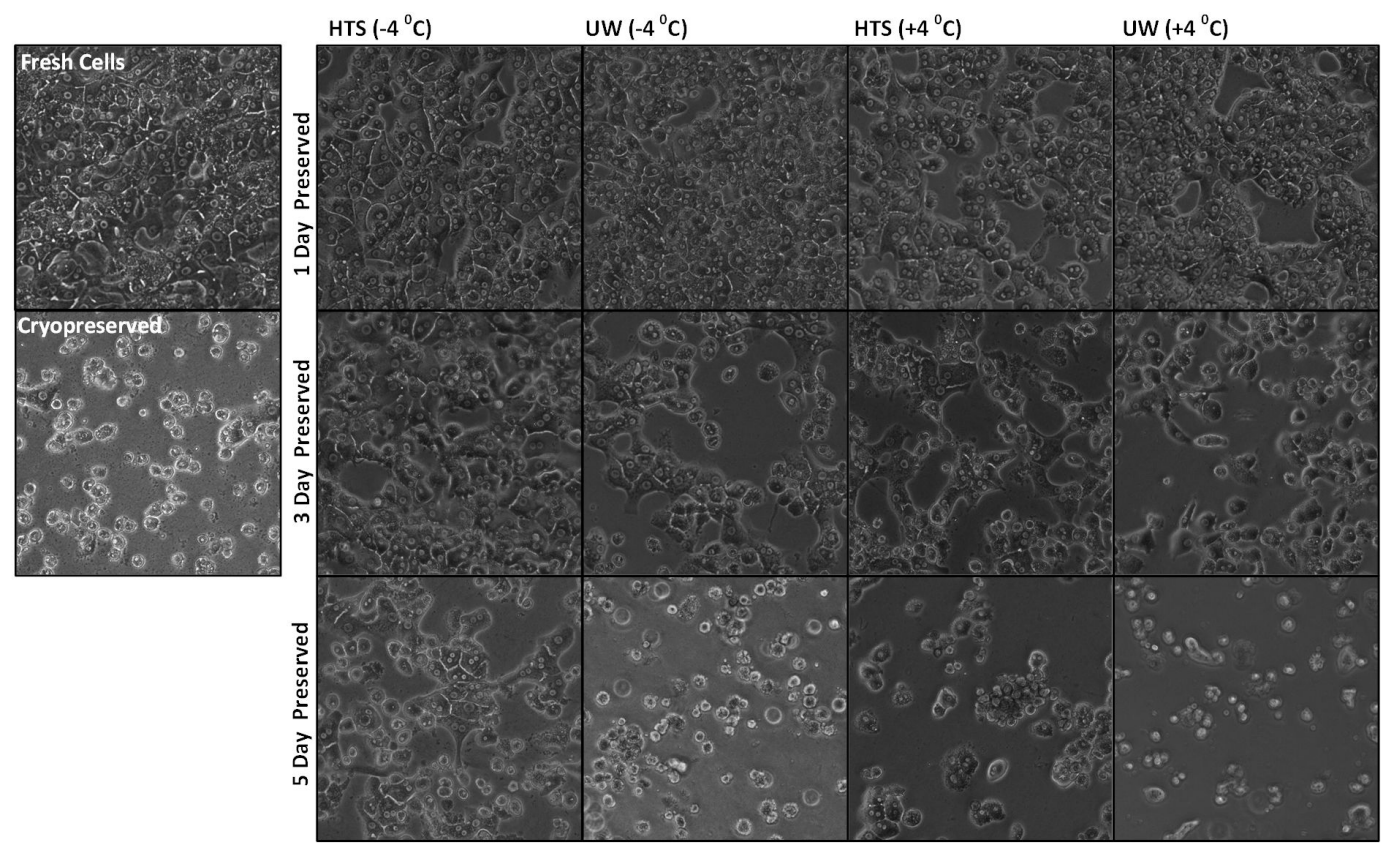
Comparison of the supercooling preservation of rat hepatocytes with other modalities in plate culture. Representative images of cell cultures of preserved and control groups after 48 hr of culture (24 hrs after the top collagen gel application).

**Figure 4:**
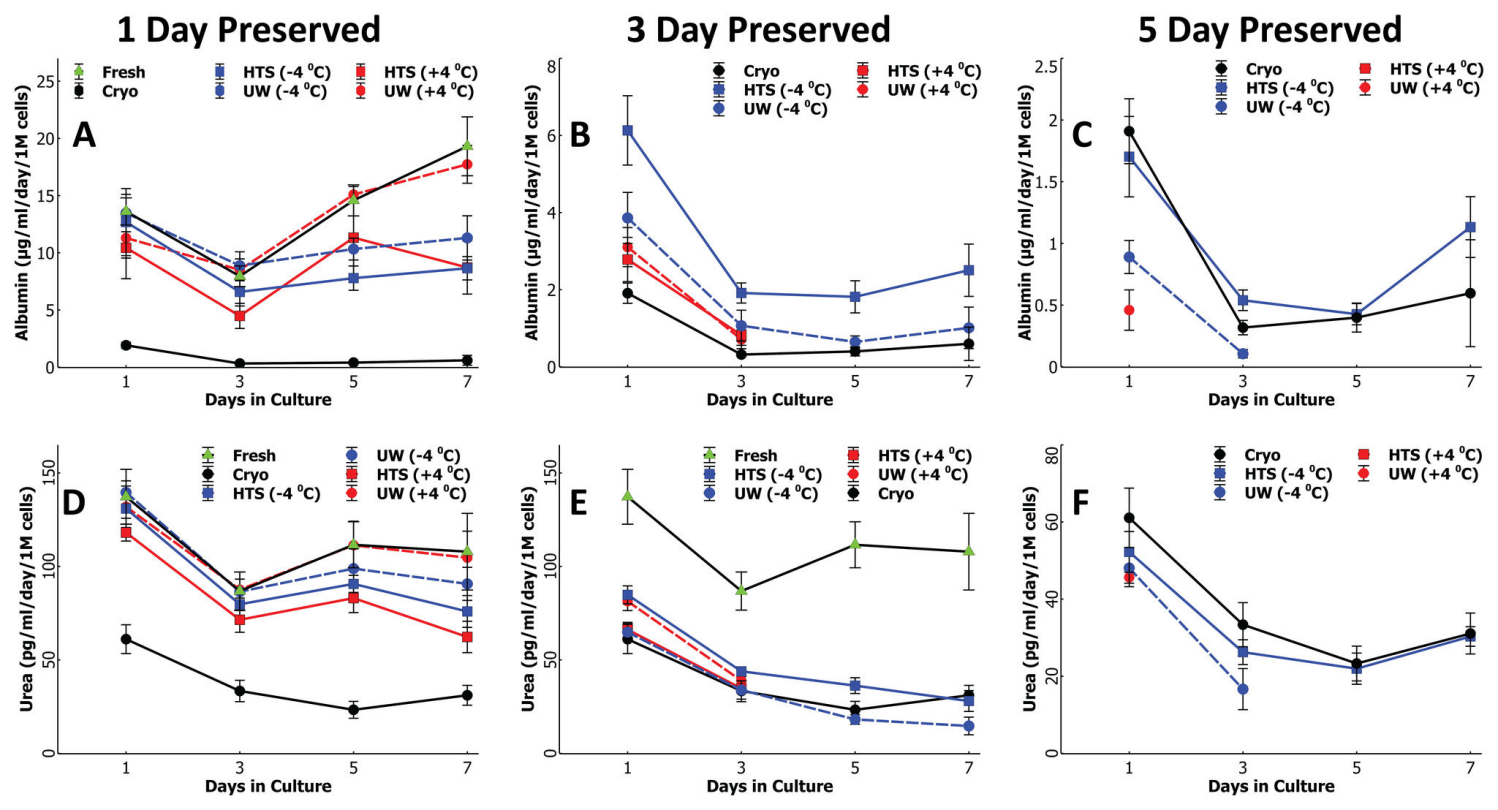
Comparison of the supercooling preservation of rat hepatocytes with other preservation modalities in long term plate culture. Albumin secretion of cells preserved for A) 1 Days B) 3 Days and C) 5 Days at -4^o^C or +4^o^C compared to fresh and cryopreserved cells. A) Cells preserved for one day in UW at +4^o^C show statistically higher (p<0.05) albumin secretion in culture beyond 4 days of culture compared to other preserved groups; this is similar to fresh cells. B) Cells preserved for 3 days in HTS at -4^o^C produces statistically higher amount of albumin compared to all other preserved groups yet significantly lower than fresh cells. C) Cells preserved for 5 days in HTS at -4^o^C produces statistically similar amount of albumin compared to cryopreserved groups which is significantly lower than fresh cells. Urea secretion of cells preserved for D) 1Days E) 3 Days and F) 5 Days at -4^o^C or +4^o^C compared to fresh and cryopreserved cells. D) All nonfrozen groups show statistically similar urea production in culture for 5 days which is also similar to fresh cells yet significantly higher than cryopreserved cells. E) Cells preserved for 3 days in HTS at -4^o^C produces statistically higher amount of urea compared to all other preserved groups yet much lower than fresh cells. F) All preserved groups show significantly lower urea production than fresh cells; cryopreserved and HTS -4^o^C group show statistically similar production for all days in culture.

In Figure 3 we present phase contrast microscopy images (48 hrs following plating and 24 hrs after top collagen gel application) of cells preserved for 1 day (first row), 3 Days (second row), and 5 Days (third row) along with fresh and cryopreserved cells (first column); this accompanies the analysis of the albumin and urea secretions (up to 7 days) for the same groups in Figure 4. The microscopic images show that 1 day preserved cells (Figure 4, first row) in either solution (HTS and UW) at either temperature (±4^o^C) show very similar morphology and attachment density comparable to fresh cells. This is confirmed by albumin secretion (Figure 4 A) and urea excretion (Figure 4 D) which are statistically same (p>>0.05) as the fresh culture for the first 3 days. The cryopreserved cells produce remarkably low secretions and excretions, and display low attachment and bad morphology. Although all of the non-frozen preserved cells show stable and high secretions, the group preserved at +4^o^C in UW shows statistically better secretions in culture beyond 5 days of culture.

By the third day of preservation, the best performing group in terms of albumin secretion (Figure 4 B) becomes cells preserved in HTS at -4^o^C (p <<0.05 compared to all other groups for all culture days) yet there are no discernible differences in terms of urea secretions (Figure 4 E); however, both albumin and urea secretions have reduced drastically from 1 day to 3 day preserved cells. The microscopic images (Figure 3, second row) confirm this finding. By the fifth day of preservation, none of the groups produce significant amounts of albumin (Figure 4 C) and only cells preserved at -4^o^C could be cultured beyond first two days. This can be easily inferred by the state of cell cultures in Figure 3 where only cells preserved in HTS at -4^o^C show good attachment and morphology albeit at a lower density. The rest of the groups suffer from poor attachment and apoptosis/necrosis during the initial 48 hrs of culture (Figure 3, third row).

### C. Scale up of the Supercooling Protocol

From our previous analysis in suspension (Figures 1 and 2) and plated culture (Figures 3 and 4) we deduce that cells preserved in HTS at -4^o^C show the most promise for extended preservation. Nevertheless storing cells in small numbers is impractical and costly for storage, handling and transport for large scale uses, such as for cell transplantation where billions of cells are needed per use. In what follows we present the results of the successful scale-up of the supercooling preservation process in HTS solution that will remedy this shortcoming. In this scale-up process we have stored 50 million cells suspended in 10 ml of HTS solution in a common 50 ml centrifuge tube. We will refer to this scaled-up group as “big tube” (BT) and the original group that was stored in small vials as “small vial” (SV) from here on in the text.

**Figure 5:**
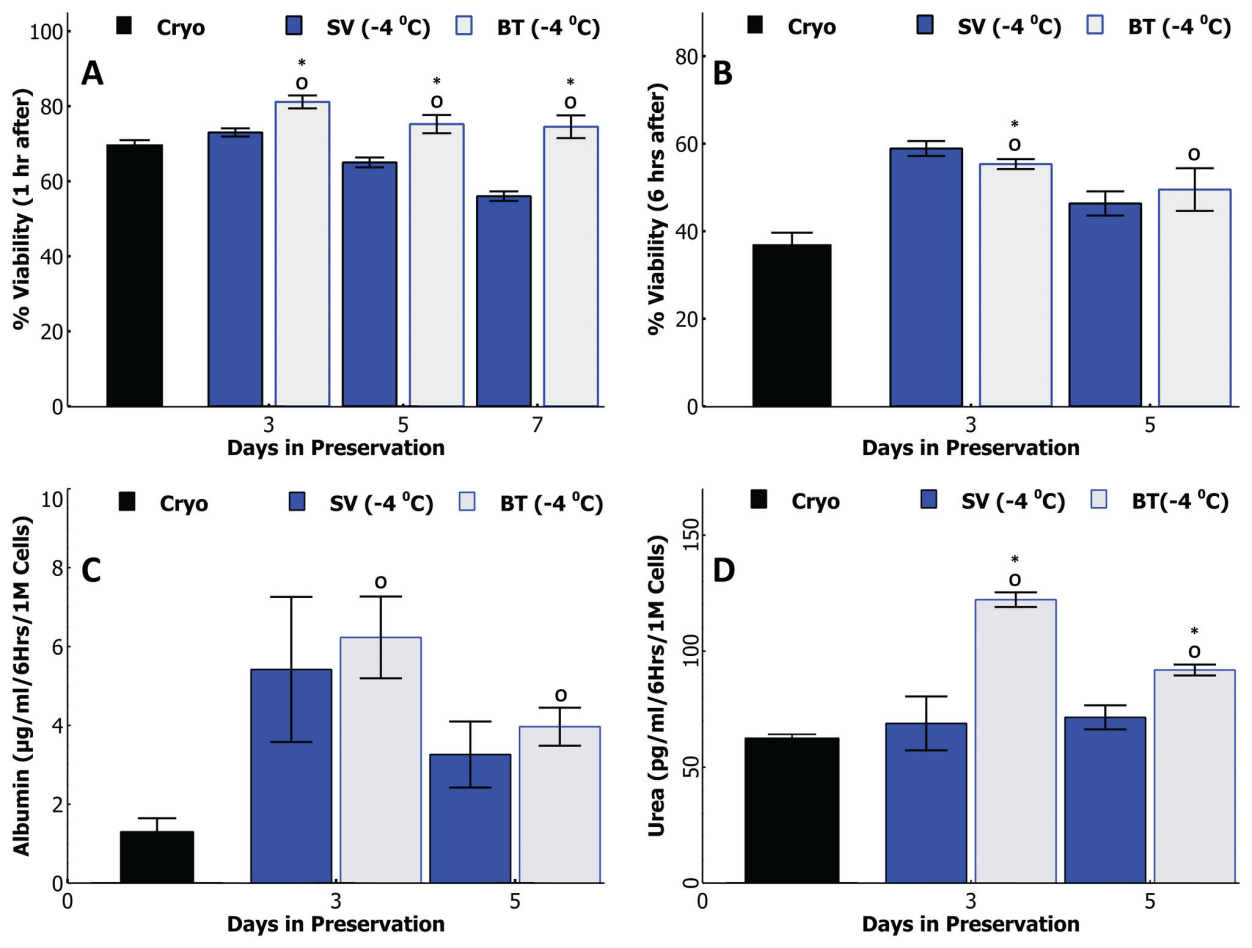
Scaling up, Comparison of cells preserved in small cryo vials (1.5M cells/vial) and big centrifuge tubes (50M cells/tube). A * and ^O^ means statistically different (p<0.05) than small vials (SV) and cryopreserved cells respectively. A) Viability of cells after 1 hour of suspension rewarming post-preservation. Cells preserved in big tubes show significantly (p<0.05) higher 1 hr viability compared to both small vials and cryopreserved cells B) Viability of cells after 6 hours of suspension culture. Cells preserved in small vials show statistically higher 6 hr viability after 3 days of preservation, cells preserved in small vials and big tubes show similar viabilities that is statistically better (p<0.05) compared to cryopreserved cells. C) Albumin production by cells during 6 hours of suspension culture D) Urea production by cells during 6 hours of suspension culture; cells preserved in big tubes show statistically higher urea production (p<0.05) on both days.

The cells in big tubes along with small vials were preserved for 3, 5, and 7 days at -4^o^C. In Figure 5 we present the viability and functionality comparison of these two groups after 1 hour and 6 hours of suspension rewarming. Cells preserved in big tubes consistently show a statistically higher 1 hr viability (Figure 5 A) and the decline in viability for this group is even slower compared to the small vials. In fact, the cells preserved in big tubes show a significantly higher viability (~75%) compared to the cryopreserved cells (~70%) (p<0.05) even at the end of a week. After 6 hours of rewarming both big tube and small vial preserved cells show almost identical viabilities (Figure 5 B) significantly higher (p<0.05 for both) than the cryopreserved group. Albumin production (Figure 5 C) via 6 hours suspension studies reveal similar functionality for both big tubes and small vials. Big tube preserved cells do show significantly better (p<0.05) urea genesis (Figure 5 D) after both 3 and 5 days preservation in the 6 hour suspensions culture.

**Figure 6:**
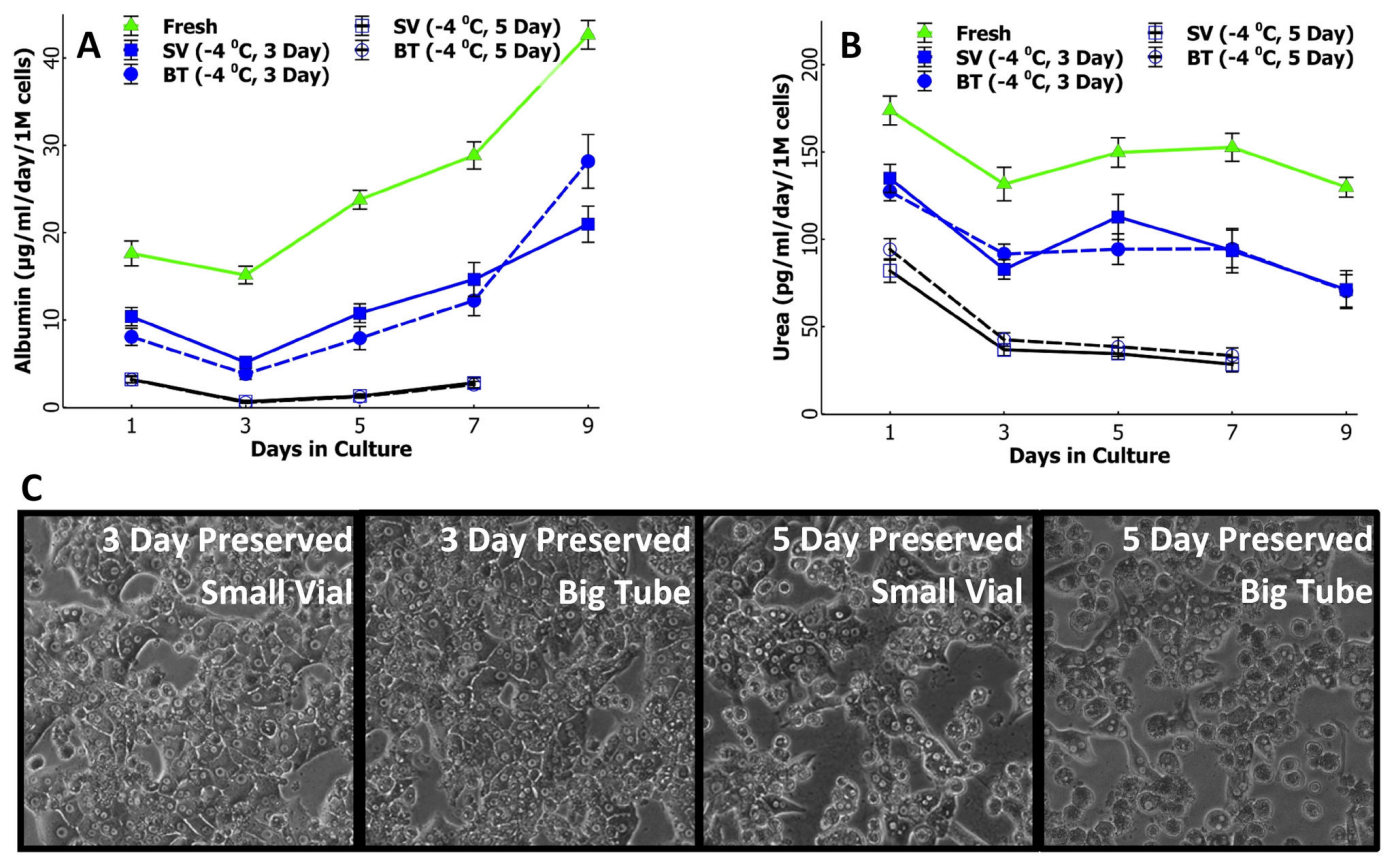
Scaling up, Comparison of cells preserved in small cryo vials (1.5M cells/vial) and big centrifuge tubes (50M cells/tube). A) Long term albumin production of 3 and 5 day preserved cells in sandwich plate culture; both groups show statistically similar (p>>0.05) albumin secretion. B) Long term urea production of 3 and 5 day preserved cells in sandwich plate culture; both groups show statistically similar (p>>0.05) urea production C) Representative phase contrast images of cells after 2 days of plating.

In addition to suspension culture we also compared the long-term plate culture performance of the big tubes against the small vials; here we used 6 well plate collagen sandwich format as opposed to 24 wells due to the availability of a large number of cells and relatively smaller experimental group. In Figure 6 A and B we show the albumin and urea production by cells preserved in big tubes or small vials for 3 and 5 days. Both preserved groups show statistically equal functionality in culture after both 3 days and 5 days of preservation which is exceedingly better compared to all the other groups in Figure 3. While 3 day preserved cells recapture almost 50% of freshly isolated cells functional capacity, for 5 days preserved cells this drops to about 10-15%; Representative phase contrast microscopic images (Figure 6 C) show that cells preserved in either big tubes or small vials have similar attachment and morphological characteristics confirming the secretion results.

### D. Membrane Damage Assessment using Scanning Electron Microscopy

**Figure 7:**
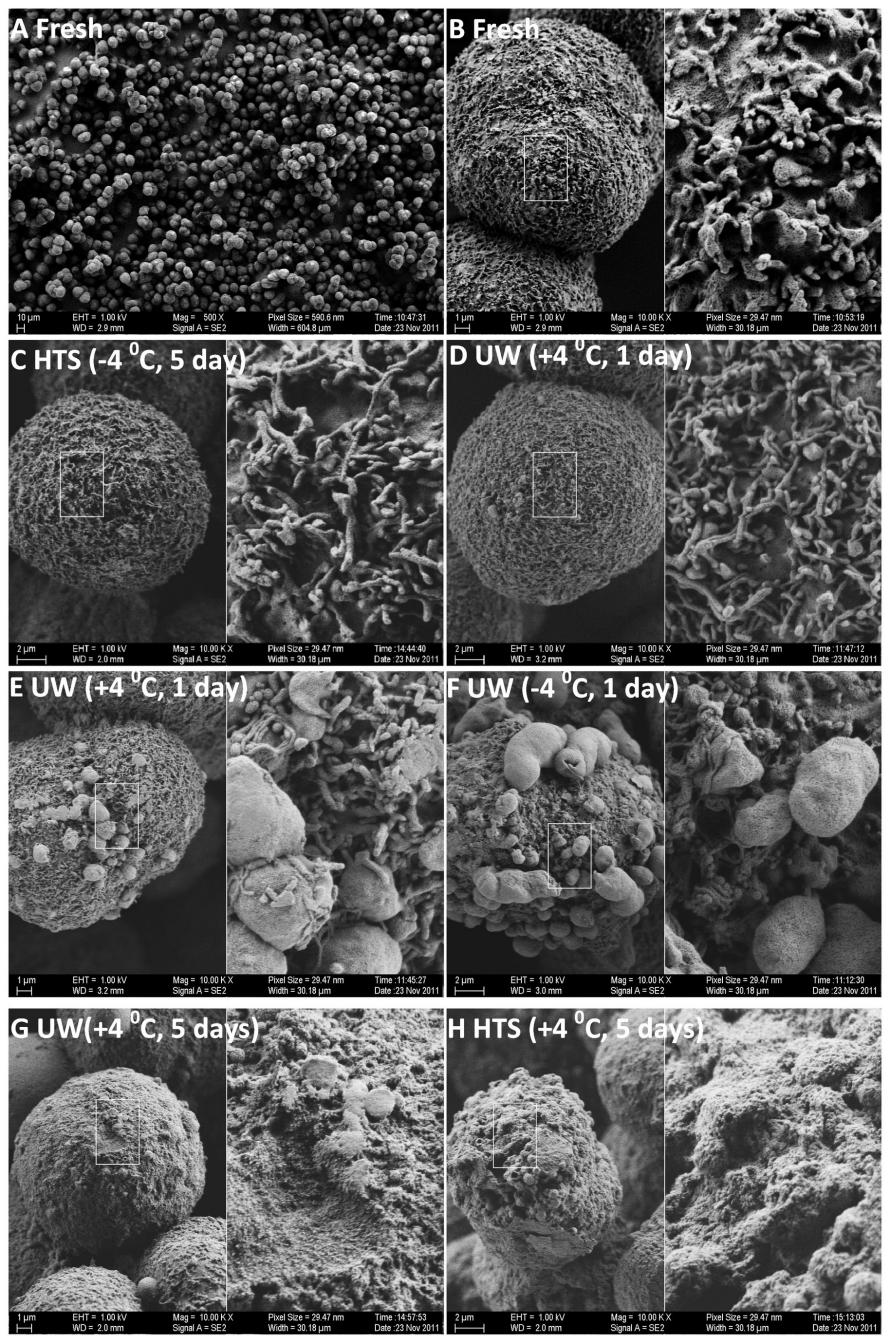
Representative SEM images of fresh and preserved cells. A) Wide angle view of freshly isolated cells B) High magnification view of a freshly isolated cell. C) Intact preserved Cell D) Intact preserved cells with very minor blebbing E) Mild and F) severe blebbing damage on preserved cell membranes G) and H) Complete loss of ultrastructure on cell membranes which occur only on cold stored (+ 4^o^C) cells beyond 3 days.

In an effort to understand cell membrane injury to the hepatocytes during the different preservation processes we have conducted scanning electron microscopic imaging of all groups for up to 5 days of preservation. In Figure 7 we present representative images of this study corresponding to the different modes and extent of damages. Figure 7 A shows a wide angle view (500 X) of freshly isolated hepatocytes; in Figure 7 B we present two levels (1000 X (left) and 5000 X (right)) of magnification on these fresh cells to show the overall intact membrane structure and the microvilli as a reference for all preserved groups. In Figure 7 C–F) we present images of cells preserved under different conditions with different damage patterns. In C) we show an intact cell with no visible membrane damage whereas in D) we present a cell with very minor blebbing. In E and F we show increasing levels of blebbing, which are also visible even at very low magnifications. In the last row (G-H) we depict a different mode of damage where the ultrastructure (microvilli) on the cell membrane are completely lost; we have observed this mode of damage only in the cold stored (+4^o^C) cells beyond 3 days of storage and this constitutes the major mode of damage for those groups.

We also present high and low magnification images of both healthy (undamaged) and damaged cells for each group in supplemental data (Figure S1-6) for further analysis by interested readers. Our observations indicate the fraction of damaged cells to be loosely correlated with the viability measures presented in Figure 1; this can be, inferred via the low magnification images of different groups (Figure S1-3). Nevertheless, such low magnification images only allow the identification of mild to major blebbing (Figure 7 E-F) and one would completely miss the ultrastructure loss which constitutes the major mode of damage in the cold stored (at +4^o^C) cells (beyond 3 days); therefore one should exercise caution when viewing these low magnification images.

## Discussion

We hypothesized that lowering the temperature below CS (+4^o^C) should prolong preservation times by slowing down of cellular metabolism. In fact most enzymes of normothermic animals are expected to show a 1.5-2 fold decrease in metabolic activity for every 10^o^C decrease in temperature [2,24,25]. We have, indeed, shown in Figure 1 A and B that the ATP expenditure, for cells preserved at -4^o^C is roughly 10 times slower compared to +4^o^C; this was beyond the expectation based on pure kinetics [2,24,25]. However, as we have seen in Figure 1 A, this dramatic slow down of metabolism (assessed by ATP retention), does not continue at lower temperatures (beyond -4^o^C); in fact ATP expenditure is much higher at -10^o^C compared to -4^o^C. We hypothesize that this increase in ATP loss at -10^o^C might be due to membrane related damages as well as an apoptosis cascade during storage; further research is needed here.

Based on this evidence and along with the high supercooling success rate (i.e low freezing probability) at -4^o^C; our next corollary was that this optimum supercooling temperature would extend preservation times dramatically beyond current CS approaches, while avoiding injuries caused by phase transitions that complicate the cryopreservation process. Throughout the results section we have tested our hypothesis h when the preserved cells are assessed for their a) viability (1 & 6 hours), b) function in short term suspension culture (6 hours) and long term plated culture (7 days) c) enzymatic activity d) membrane integrity via SEM imaging. Results show that preserved in HTS at -4^o^C retain their viability and function much better when compared to the gold standard CS preservation at +4^o^C in UW.

A secondary question that arose during our studies was if there are any discernible differences between the two commercially available solutions for use in supercooling preservation. While neither solution is specifically designed for SCP, evidence throughout the rest of our results show that HTS-FRS is a significantly better preservation solution at -4^o^C for storage (≥ 3days) and this combination (HTS at -4^o^C) is the optimum among all SCP and CS groups. However, we have also found that UW performs better at +4^o^C compared to HTS beyond the third day of storage.

This is a curious reversal of roles between the two solutions. To the best of our knowledge apart from the differences in ionic concentrations and use of different buffers and impermeants, both solutions focus on preserving the intracellular balance; both solutions contain lactobionate which has been implied as the key component to avoid injury to liver cells in cold storage [26,27]. While the UW solution is rich in potassium (K+), the HTS-FRS solution is rich in sodium (Na+); nevertheless recent findings [26,27] suggest that this is not a distinguishing feature for long term hypothermic storage. Similarly although the osmotic pressure of the two solutions differ slightly (~350 mOsm for HTS-FRS and ~320 mOsm for UW) and this can cause a small variation in cell shrinkage during preservation, this effect has also been found to have minimal impact on cold storage of rat hepatocytes [27].

An important difference in the formulations of the two preservation solutions is the way they handle the reactive oxygen species generated during the preservation. While the UW solution contains both glutathione and allopurinol to combat intracellular ROS, HTS-FRS solution includes glutathione as well as a very potent general antioxidant, “Trolox”, in its formulation. Trolox is also known to have a significant iron-chelating capacity [28]; iron chelators have been found to be one of the key elements to enable high attachment efficiency of preserved cells after long term hypothermic storage of rat hepatocytes [27,28]. The importance of Trolox for cold storage of human hepatocytes beyond 48 hours was shown by Ostrowska and co-workers [29] where they compared HTS-FRS against UW and HTS-Base (without Trolox) and found that HTS-FRS was the only solution to prevent damages beyond 48 hours. While ROS generation during short static cold storage at +4^o^C has been shown to be low [30] and the lipid peroxidation to be slow [31], similar data does not exist for long storage during cold storage or supercooling. Based on this discussion we hypothesize that the accumulation of ROS and lipid peroxidation might be overwhelming the cellular protective mechanisms during long-term storage at the SCP temperatures and that an external potent antioxidant such as Trolox might be the key prolong successful preservation. A comparative assessment of ROS generation and lipid peroxidation at SCP temperatures with the two solutions should reveal whether an antioxidant is indeed a necessity for SCP; this is an ongoing work in our laboratory.

The cellular membrane is prone to injury during rapid cooling and phase transitions (cryopreservation) [32,33] or prolonged cold exposures (CS) [4,34]. SEM imaging of SCP hepatocytes reveal that membrane injury is also present during supercooling preservation; however, whether this damage is the result of direct lipid peroxidation or phase transition or an internal cascade manifesting (apoptosis, ion imbalance) itself as eventual blebbing is unclear. Notably, loss of ultrastructure (ie surface proteins) occurred only on cells that are preserved at 4^o^C long term, whereas SCP group retained their surface ultrastructure throughout the whole preservation period, in correlation with enhanced function and preservation of hepatocytes after SCP in HTS. However, continued presence of membrane injury indicates this is a profitable direction for further improvements, such as membrane stabilizers.

Storing cells in 1.5 million batches in small vials, although successful, is not practical for handling large quantities and it would prove to be expensive for large scale use cases such as cell transplantation or high throughput screening schemes. Therefore a crucial improvement for the SCP modality was the scale-up that we have presented successfully in Figures 5 and 6. The scaled-up preservation in big centrifuge tubes (50 million cells/tube) was equal or better in terms of both viability and functionality. this scale-up not only reduces the consumables, which are relatively expensive, but also considerably decreases the handling and processing time when a large number of cells are required. The difference in cell quality between small vials and big tubes, while small, is interesting. The elucidation of mechanistic differences during preservation in these two modalities will be performed as part of our future research with human hepatocytes where preservation of large quantities is relatively more important.

We see in our own results that there is a significant decline in viability from 1 hour to 6 hours of rewarming suspension culture; more dramatic for some modalities than others. Our screening studies along with literature data [26,27] show that there is also about a 10 percentage point decline from 0^th^ to the 1^st^ hour. These differences between 0, 1 and 6 hour viabilities might be due to a fraction of the cells in the early phases of apoptosis that still have intact membranes; this is also coupled with the fact that there is usually considerable amount of ROS generated during rewarming [30] for cold stored cells leading to further cell death. For this reason we assert that viability studies immediately after preservation should in general be avoided, and at least a six hour suspension should be performed for assessment of viability.

## Conclusion & Future Outlook

In this paper we have presented a comparative study of several non-freezing preservation methods using different commercially available preservation solutions (HTS and UW) at a range of different temperatures and we also compared these to freshly isolated cells and cryopreserved cells. We have found that cells preserved at -4^o^C in HTS solution - a supercooling preservation protocol - show the highest viability as well as functional capacity both in short term suspension culture and long term plate culture, dramatically better than cryopreserved cells within a preservation duration up to 5 days. This supercooling preservation protocol was further improved by scaling it up to 50x10^6^ cells in big centrifuge tubes with outstanding viability and similar or better functionality compared to low density preservation (small vials). While the results need to be confirmed with human hepatocytes, the viability after preservation are very similar across hepatocytes species and we do not expect major differences in results.

The SCP protocol presented increases the viable preservation duration of hepatocytes up to 7 days, while avoiding the highly disruptive freezing process. In the short-term, such a non-freezing approach is highly useful for cell transplantation and bioartificial liver studies - which require a large number of cells, and cryopreserved cells are often avoided- since the donor liver, isolation process, and the patient can be at different locations, and scheduling can be optimized [35]. In fact, the increased preservation duration would enable world-wide distribution of hepatocytes, hence a donor liver in Europe could be used as a source of cells for a patient in the US, or vice-versa. In the long-term, the ability to ship tissue-engineered products or whole livers across continents would be a critical enabling technology for translation and commercialization that remains missing [36].

Although we have presented a successful supercooling preservation that extended the useful preservation time for non-freezing storage to almost a week there is still much room for improvement to the method we describe in here. Since in this study we did not further supplement HTS at any step of preservation, results could be improved significantly by optimizing for supercooling. Some of the possibilities include antioxidants for treatment before, during, and after preservation, as well as membrane [37–40] and cytoskeletal stabilization [41,42] strategies. In addition to these traditional additives, newer strategies such as membrane composition modification using lipid exchange [33,43,44] can also provide alternate routes towards both understanding and minimizing preservation damage. Extending the supercooling preservation times beyond a week and establishing a deeper understanding of sub-zero non- freezing biology in a mechanistic way will be an important step forward that can dramatically alter the way we approach dissemination of tissues and tissue engineered products, and organ banking and transplantation.

## Materials and Methods

The Subcommittee on Research Animal Care, Committee on Research at the Massachusetts General Hospital, approved the experimental protocols that relates to the handling and use of animals (cell isolation) in this manuscript (Permit # 2011N000111).

### A Supercooling and Static Cold Storage

#### Storage

Freshly isolated cells were used for all our cell preservation studies. After the isolation, cells in DMEM solution were kept on ice and then resuspended, in either HTS (HTS-FRS solution, Biolife Solutions) or UW (CoStorSol, Preservation Solution Inc.) solution, at a density of 1.5X10^6^ cells/ml. The suspensions were then aliquoted (1.0 ml) into 1.5 ml Nalgene cryovials which were kept on ice throughout. These vials were quickly transferred onto a plastic vial holder and then to their respective storage units at -4^o^C (for SCP) and +4^o^C (for CS). These constitute the 4 non-freezing experimental groups with cells stored a) in HTS at -4.4 b) in HTS at + 4^o^C) c) in UW at -4.4^o^C d) in UW at 4^o^C. Additional subzero temperature groups (-7^o^C and -10^o^C) were also used for initial screening experiments. For the subzero temperatures we employed a portable temperature controlled freezer (Engel MHD-13, Engel-USA Inc.) whereas for the 4^o^C stored groups we used a temperature controlled cold room. For the scale up studies using the HTS solution we used 50ml centrifuge tubes as the storage container with 10 ml of 5x10^6^ cells/ml suspension (50x10^6^ cells/centrifuge tube). 50 ml tubes were chosen since re-suspending large cell pellets in 50ml tubes put less stress on the cells.

#### Rewarming

3 cryovials from each group were transferred onto ice and then centrifuged at a temperature of 4^o^C and speed of 300 rpm after 1, 3, 5 and 7 days of storage. The cells were washed and the medium was then replaced with ice-cold C+H media and the vials were kept at room temperature for 3-5 minutes. The cells were then placed on a rototorque, at the lowes rotational speed, in a warm room (37^o^C) and kept in suspension for one hour before any further analysis was performed.

### B. Cryopreservation

#### Storage

Cells were resuspended in HTS-FRS solution containing 10% DMSO (v/v) with a final concentration of 1.5 X10^6^ cells/ml and kept on ice; 1 ml aliquots were transferred to cryovials and again kept on ice. The programmable controlled rate freezer, KRYO560 (Planer Co, UK), was cooled down to 10^o^C and then the vials were transferred on to an in-house built vial holder. The cells were cooled down to -4^o^C at a rate of -1^o^C/min. At this temperature manual ice crystal seeding was initiated by a cotton swab that was dipped in to liquid nitrogen. The freezer was then held at -4^o^C for the next 10 minutes after which they were cooled down to -80^o^C at a rate of -1^o^C/min and held there. To end the cryopreservation protocol the vials were immediately transferred to a dewar filled with liquid nitrogen and then to a larger cryo tank kept between -150 to -170 ^o^C.

#### Rewarming

3 cryovials of cryopreserved cells were transferred immediately from the cryo-tank into a water bath kept at 37^o^C; the vials were gently shaken until all the crystals disappeared and a clear solution was observed in the vials. The cells were washed and the medium was then replaced with C+H media at room temperature and kept at room temperature for 3-5 minutes. The cells were then placed on a rototorque in a warm room (37^o^C) and kept in suspension for one hour before any further analysis was performed.

### C. Plate Cell Culture

Cells were cultured up to a week using 24 well or 6 well tissue culture plates; collagen sandwich method [45–47] was used. Basically culture plates were coated with a collagen gel an hour prior to cell culture and the cells were then introduced into wells as a suspension (0.25X10^6^ and 1X10^6^ live cells /well for 24 and 6 well plate wells respectively). Upon the adherence of cells on to the collagen gel (~ 4hrs later), dead cells were washed away and the media was replenished. A second layer of collagen gel was deposited onto the cells 24 hrs after their initial deposition. Media was sampled daily and stored at -80^o^C thereafter for further analysis.

### D. Suspension Culture

Cells that were placed in C+H media during the initial phase of rewarming were kept suspended, on a rototorque, for 6 hours followed by a viability analysis. Cells were than centrifuged at 500 RPM upon which the supernatant media were withdrawn and stored at -80^o^C until further analysis.

### E. Culture Imaging

Cultured cells were imaged periodically using a Zeiss Axiovert 200 M microscope using phase contrast microscopy; digital images were captured.

### F. Suspension Live/Dead Analysis

Suspension viability analyses were conducted after either 1 hour or 6 hours of suspension culture; we avoided a viability assessment immediately after rewarming since our own experience as well as the literature [26,27] show the immediate viability assays to overpredict viability by about 10-20%. Basically 50 µL from each cell suspension was sampled after the solution was gently resuspended. 10 µL of Trypan blue and the sample was mixed in a 96-well plate well; after waiting for a minute this mixture was transferred on to a hemacytometer and the live (bright/white) and dead (blue stained) cells were counted from 4 quadrants.

### G. ATP Analysis

3 cryovials (1.5X10^6^ cells/vial) from each group were transferred onto ice from storage and the cells were centrifuged down at 300 rpm. The preservation medium was aspirated and the cells were washed with ice-cold PBS twice; all the medium was then aspirated while keeping everything on ice. The cryovials with cell pellets were then quickly plunged into liquid nitrogen to flash-freeze the cells. The cell pellets were kept at -80^o^C until performing the ATP analysis using ApoSensor ATP analysis Kit (Biovision Inc.). At the time of analysis, each vial was transferred onto dry ice and handled one by one as follows: the cell pellet was resuspended in 1ml PBS; a 25 µL sample was withdrawn from this solution and mixed with 500 µL of oligonucleotide releasing buffer (NRB) from the kit. This solution was then diluted 1:4 (final solution volume of 110 µL) in a luminometer test tube and the luminescence was measured using a FB-12 Luminometer (Bertholds Inc); the peak reading within a 60 second window was recorded; this value was then converted into corresponding ATP concentration in the sample using a standard curve constructed simultaneously.

### H. Suspension EROD Analysis

We have measured the EROD (ethoxyresorufin-O-deethylase) activity of both experimental and control groups using a suspension; the assay measures the CYP1A mediated deethylation of the substrate 7-ethoxyresorufin to form the product resorufin [48]. This assay serves as a general indicator of phase I enzymatic activity in our study. The procedure is as follows: after cells were rewarmed in the C+H media for an hour; they were spun down at 300 rpm and then resuspended in PBS to wash of the remaining C+H. This was followed by a second centrifugation at 300 rpm, PBS was replaced with Williams- *E* *medium* (Sigma) free of phenol red and glucose; cells were resuspended and the suspension were transferred from the Nalgene cryovials to black/non-transparent Eppendorf tubes. Each tube was injected with a 20 µL of 6mM dicumarol solution to inhibit any phase II metabolic activity; the suspension were then kept on a rototorque at 37^o^C for the next 20 minutes. At this point 10 µL of ethoxy-resorufin substrate was introduced into each suspension; the suspension was mixed by gentle shaking and a 50µL sample was withdrawn after letting the cells settle down in the solution –this sample constitutes the 0^th^ min sample. All the samples were kept in a separate well on a 96 well plate which was kept on ice in a non-transparent box. The vials were then placed on the rototorque, and from this point on a 50 µL sample was withdrawn from each vial every 10 minutes after letting cells settle for about 30-40 seconds before withdrawal. The withdrawal was terminated after 40 mins (5^th^ sample). 8 standards ranging from 1000 nM to 0 nM were also introduced on the assay plate and finally the fluorescence of samples were measured (ex: 530 nm, em: 590 nm) using a Synergy 2 multimode plate reader (Biotek Inc.). The sample concentrations were deduced using the standard curve and also corrected for the withdrawn amount of samples during the time series. Finally a linear regression between resorufin concentration and time – using sample points 10-40 mins – revealed the rate of resorufin production i.e the EROD activity.

### I. Albumin Analysis

We employ an in-house developed inverse competitive ELISA method to measure the amount of albumin production; media collected from plate cell culture or the suspension culture supernatant is used for the measurement. Specifically we plate 100 µl of 50 µg/ml albumin (MP Biomedicals LLC.) in each well of 96 well high-binding plates. The plates were then sealed and kept at 4^o^C over-night for the albumin to bind. The plates were then washed 4 times using PBS-Tween. 50 µL of both samples and standards (100-0 µg/ml) were then introduced as duplicates in separate wells. Albumin antibody (~2µg/ml) was introduced immediately so that the bound albumin on the plate and albumin in samples can compete for the antibodies. The plates were sealed again and incubated at 37^o^C for 90-120 mins. The plates were washed again with PBS-Tween and then developed using an OPD solution containing H_2_O_2_; the OPD solution was introduced to wells at regular intervals and the reaction was stopped with the same intervals using 8N H_2_SO_4_. The absorbance at each well were then analyzed using a Benchmark Plus microplate reader (Biorad Inc.) with dual readings at 490 and 650 nm. Standard curves were constructed for each plate and sample concentrations were calculated accordingly.

### J. Urea Analysis

We used a colorimetric urea detection assay (Stanbio Inc.). Shortly, 10 µL of both samples and standards (200-0 µg/ml) were introduced as duplicates into clear 96 well assay plates along with 150 µL of BUN reagent mixture as described in the kit. The plates were sealed tightly and then incubated at 60^o^C for 90 mins. The plates were then cooled on ice for 20 mins prior to reading the absorbance of samples at 520 and 650 nm using the Benchmark Plus microplate reader (Biorad Inc.). The standard curve was constructed and sample concentrations were calculated accordingly.

### K. SEM Imaging

Preserved cells kept at their respective storage units were transferred onto ice and then centrifuged at 4^o^C and 300 rpm. The cells were washed with PBS and the medium was replaced with 2% Glutaraldehyde in PBS. Cells were then transferred to and kept at a 37^o^C incubator for 15 minutes. The cells were then centrifuged and washed with PBS and the medium was replaced with 20% Ethanol solution. The cells were then dehydrated using a gradually increasing Ethanol concentration (50%, 70%, 80%, 90%, 95%, 100%) procedure; cells were kept in each solution for 10 minutes after which the cells were sedimented and the solution was replaced with the next solution with higher concentration. At the end of this procedure the cells were freeze-dried and the prepared for SEM Imaging via gold coating. The imaging was performed using a Zeiss Ultra Plus Scanning Electron Microscope using the SE2 detector at various magnifications.

### L. Experiments and Statistics

Experiments have been conducted with cells from at least 3 different cell isolations for each group (up to 8 times where possible) with at least 3 samples for each group in each experiment. Data on figures are expressed as the mean ± standard error (SEM); statistical significance was determined by a two-tailed student’s t-test.

## Supporting Information

Figure S1 Low Magnification SEM images showing damaged and undamaged cells preserved for 1 day in. A) fresh cells B) cryopreserved cells C) UW solution at -4^o^C D) HTS solution at -4^o^C E) UW solution at +4^o^C F) HTS solution at +4^o^C.
(TIF)

Figure S2 Low Magnification SEM images showing damaged and undamaged cells preserved for 3 days in. A) UW solution at -4^o^C B) HTS solution at -4^o^C C) UW solution at +4^o^C D) HTS solution at +4^o^C.
(TIF)

Figure S3 Low Magnification SEM images showing damaged and undamaged cells preserved for 5 days in. A) UW solution at -4 0C B) HTS solution at -4^o^C C) UW solution at +4^o^C D) HTS solution at +4^o^C.
(TIF)

Figure S4 High Magnification SEM images of cells preserved for 1 day in. A) UW solution at -4^o^C B) HTS solution at -4^o^C C) UW solution at +4^o^C D) HTS solution at +4^o^C. In each image a second higher magnification section is displayed on the right hand side corresponding to the white outlined rectangle on the left.
(TIF)

Figure S5 High Magnification SEM images of cells preserved for 3 days in. A) UW solution at -4^o^C B) HTS solution at -4^o^C C) UW solution at +4^o^C D) HTS solution at +4^o^C. In each image a second higher magnification section is displayed on the right hand side corresponding to the white outlined rectangle on the left.
(TIF)

Figure S6 High Magnification SEM images of cells preserved for 5 days in. A) UW solution at -4^o^C B) HTS solution at -4^o^C C & E) UW solution at +4^o^C D & F) HTS solution at +4^o^C. In each image a second higher magnification section is displayed on the right hand side corresponding to the white outlined rectangle on the left.
(TIF)
