# Supplementary figures and images for: Supercooling as a Viable Non-Freezing Cell Preservation Method of Rat Hepatocytes
Source: PLoS One. 2013 Jul 16;8(7):e69334. doi: 10.1371/journal.pone.0069334 (PMC3713052; doi:10.1371/journal.pone.0069334)

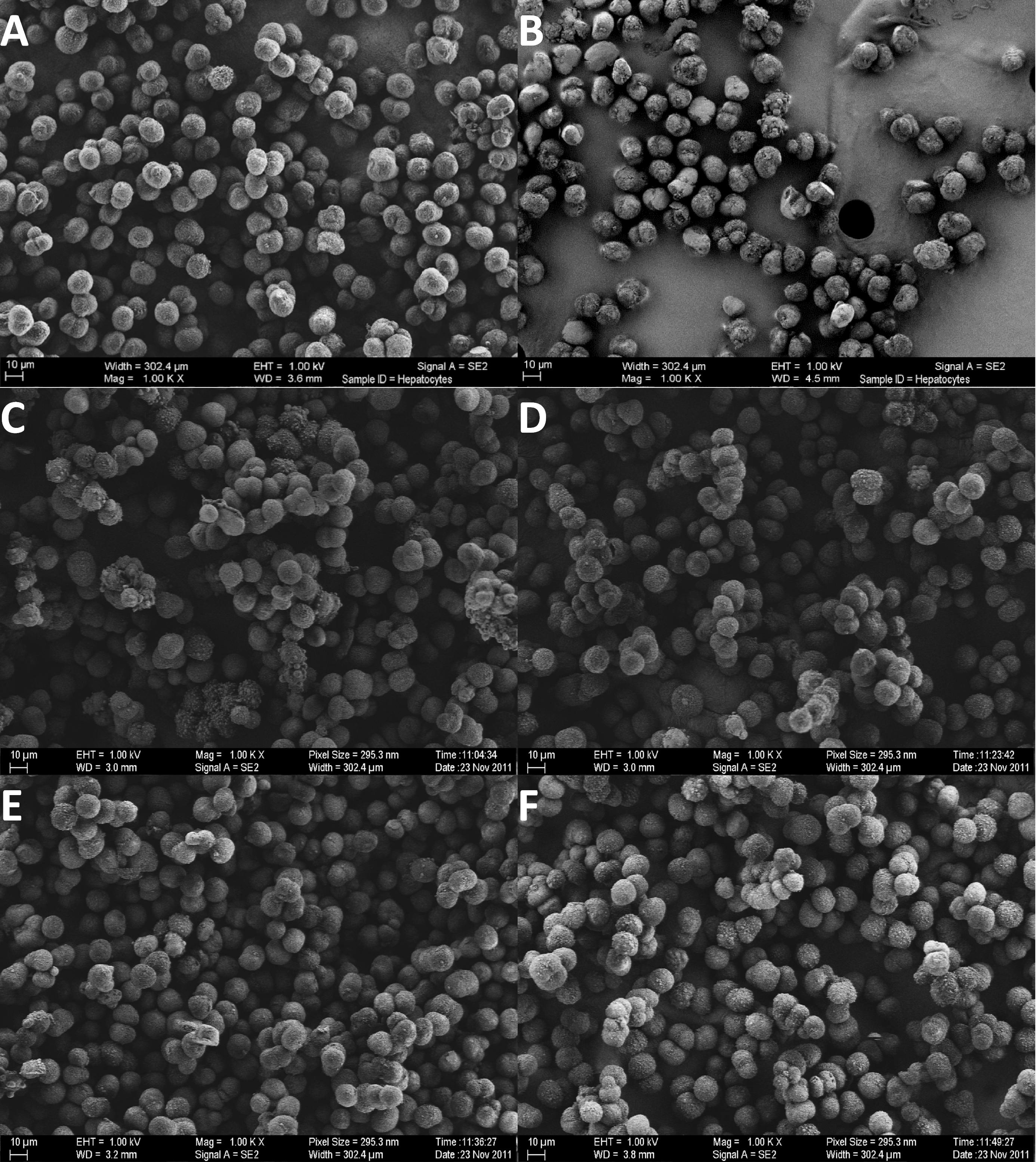

Supplement: Figure S1 — A) fresh cells B) cryopreserved cells C) UW solution at -4oC D) HTS solution at -4oC E) UW solution at +4oC F) HTS solution at +4oC. (TIF) [file pone.0069334.s001.tif]

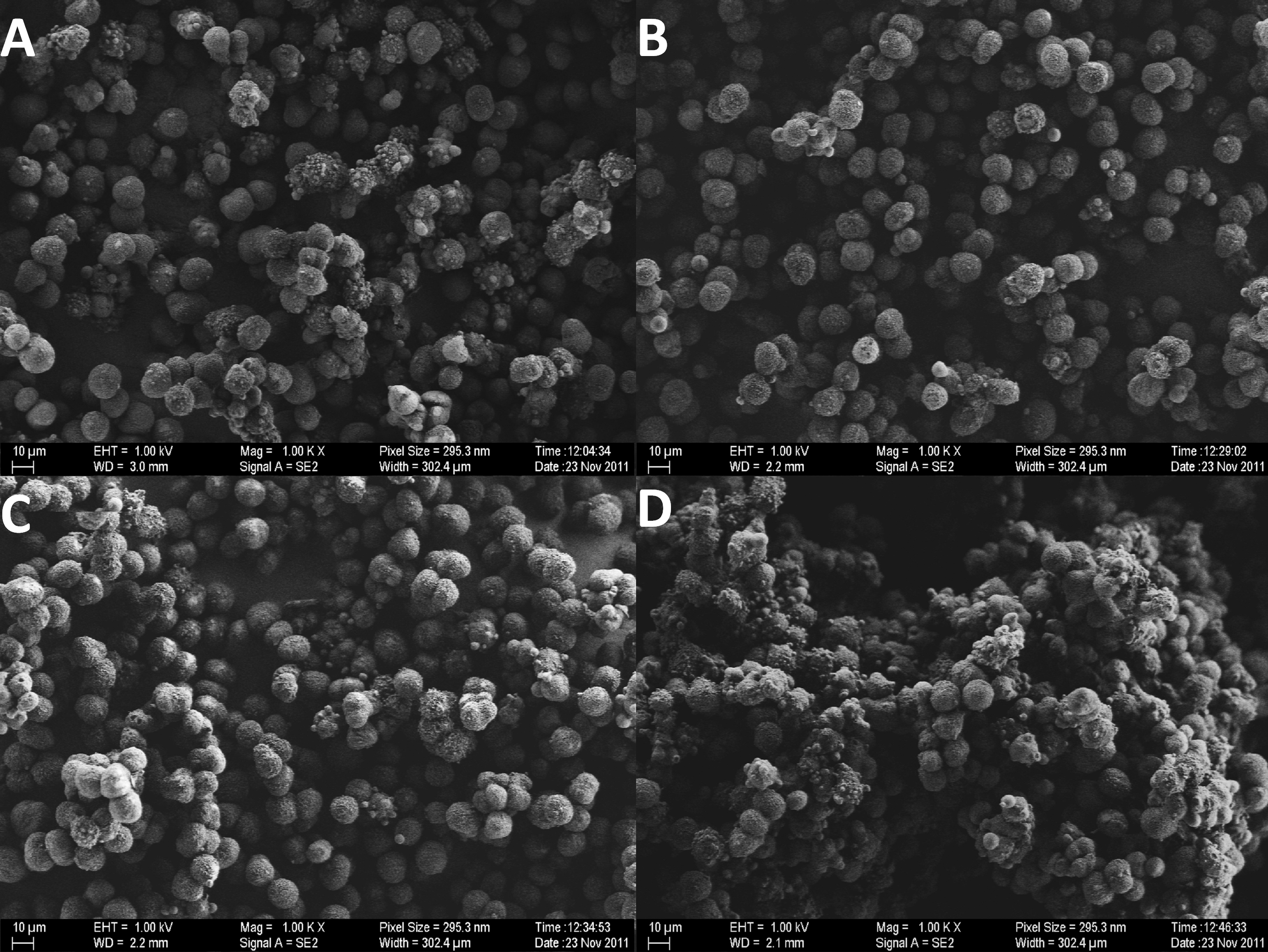

Supplement: Figure S2 — A) UW solution at -4oC B) HTS solution at -4oC C) UW solution at +4oC D) HTS solution at +4oC. (TIF) [file pone.0069334.s002.tif]

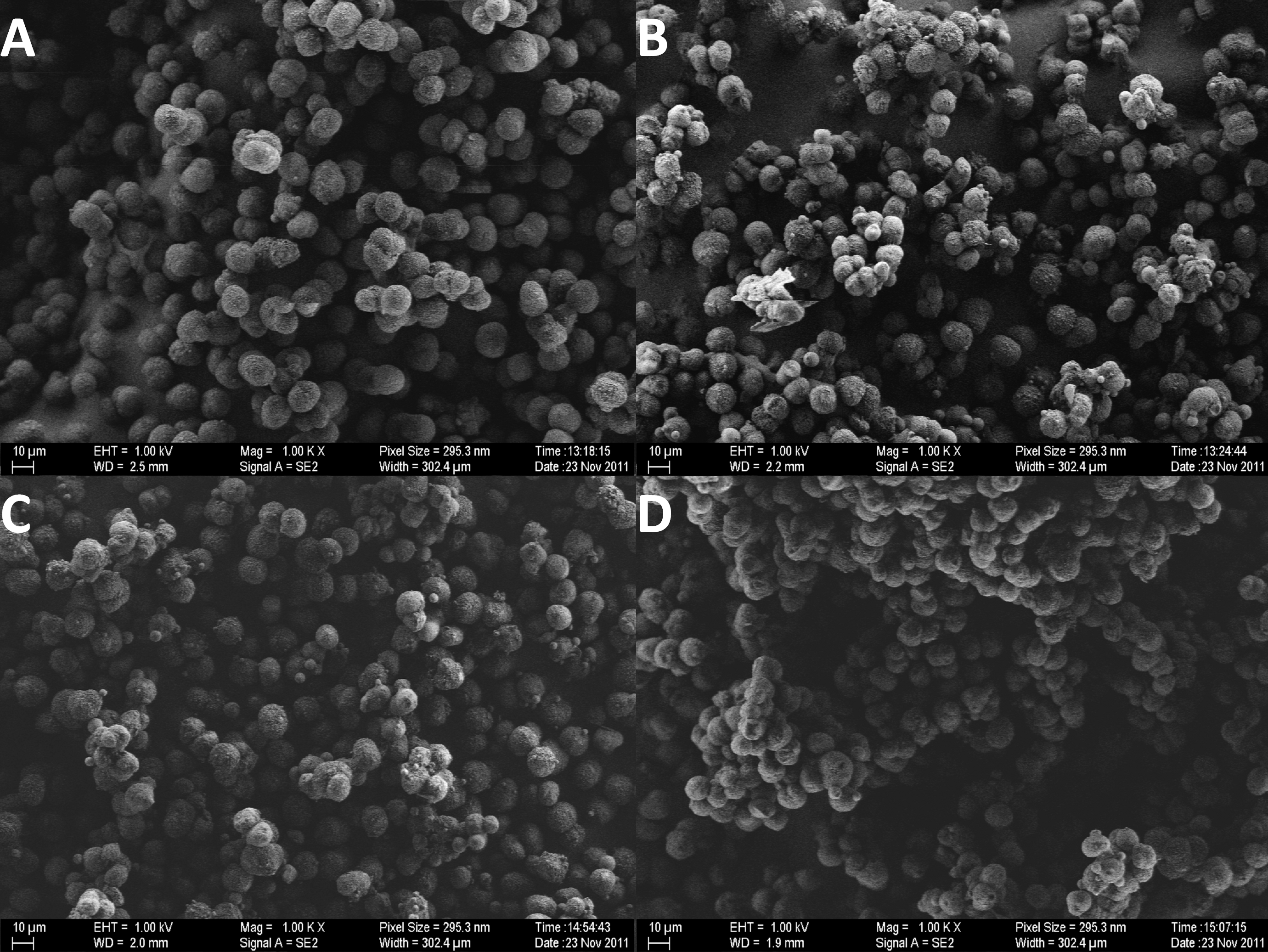

Supplement: Figure S3 — A) UW solution at -4 0C B) HTS solution at -4oC C) UW solution at +4oC D) HTS solution at +4oC. (TIF) [file pone.0069334.s003.tif]

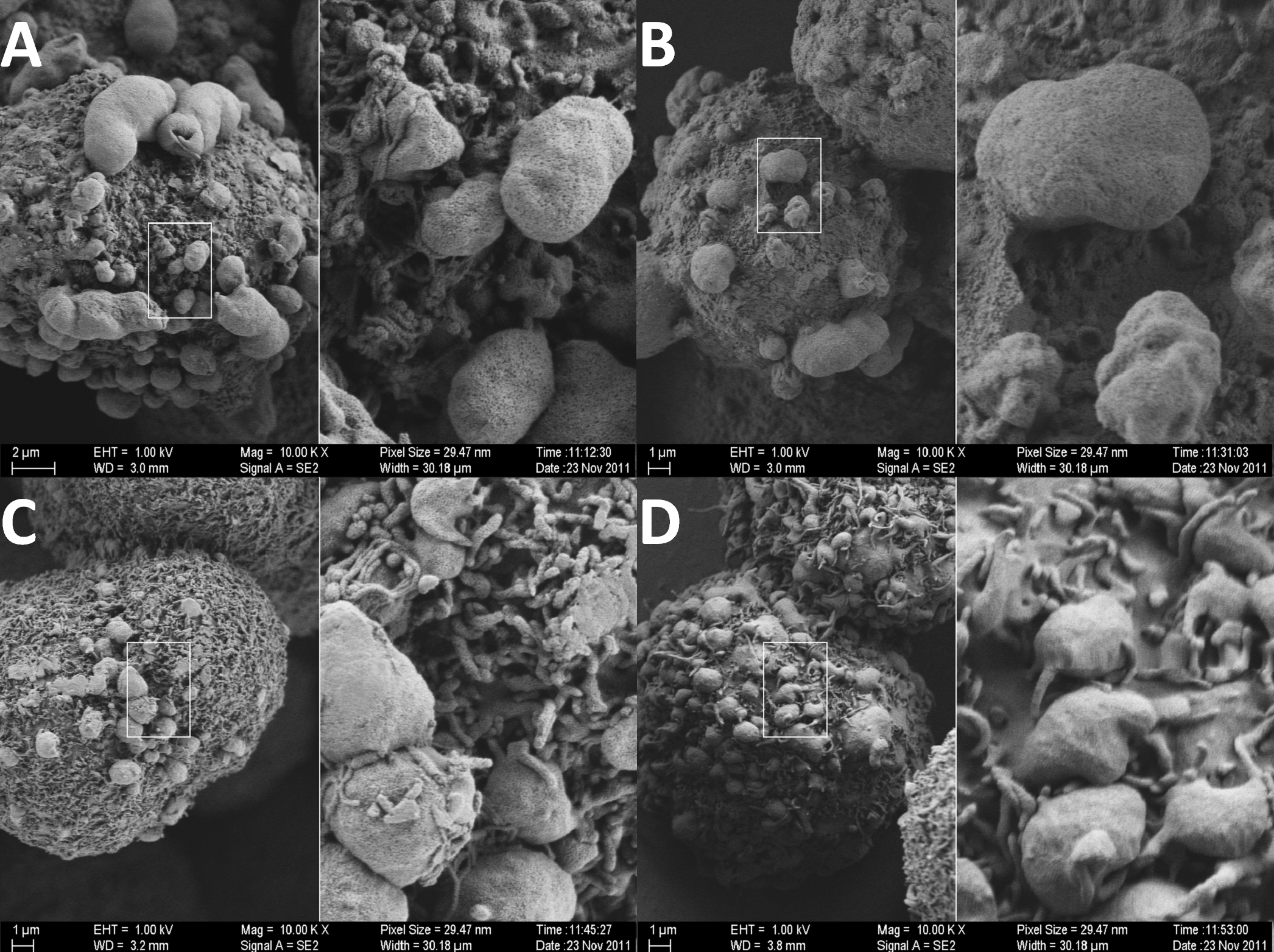

Supplement: Figure S4 — A) UW solution at -4oC B) HTS solution at -4oC C) UW solution at +4oC D) HTS solution at +4oC. In each image a second higher magnification section is displayed on the right hand side corresponding to the white outlined rectangle on the left. (TIF) [file pone.0069334.s004.tif]

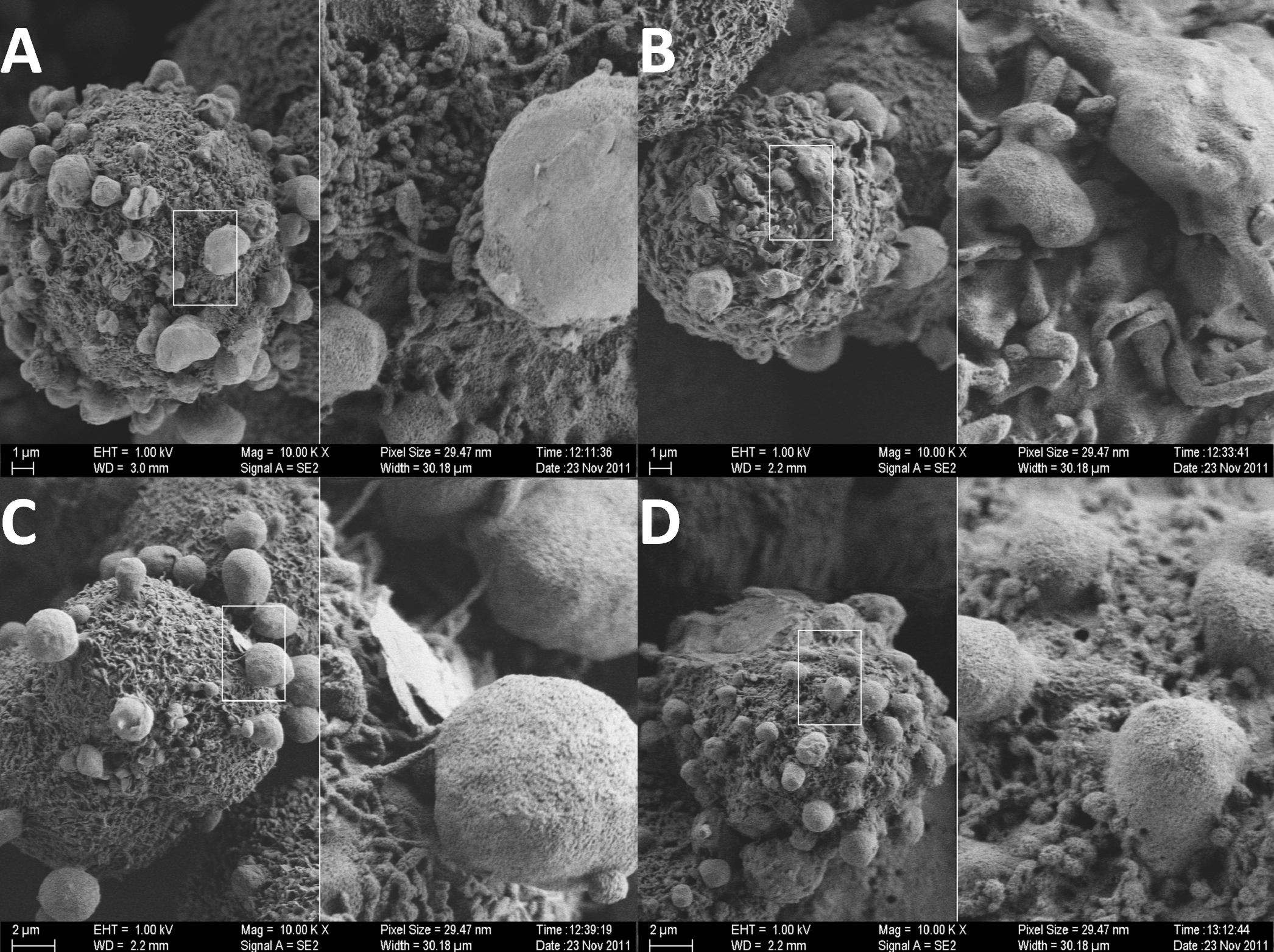

Supplement: Figure S5 — A) UW solution at -4oC B) HTS solution at -4oC C) UW solution at +4oC D) HTS solution at +4oC. In each image a second higher magnification section is displayed on the right hand side corresponding to the white outlined rectangle on the left. (TIF) [file pone.0069334.s005.tif]

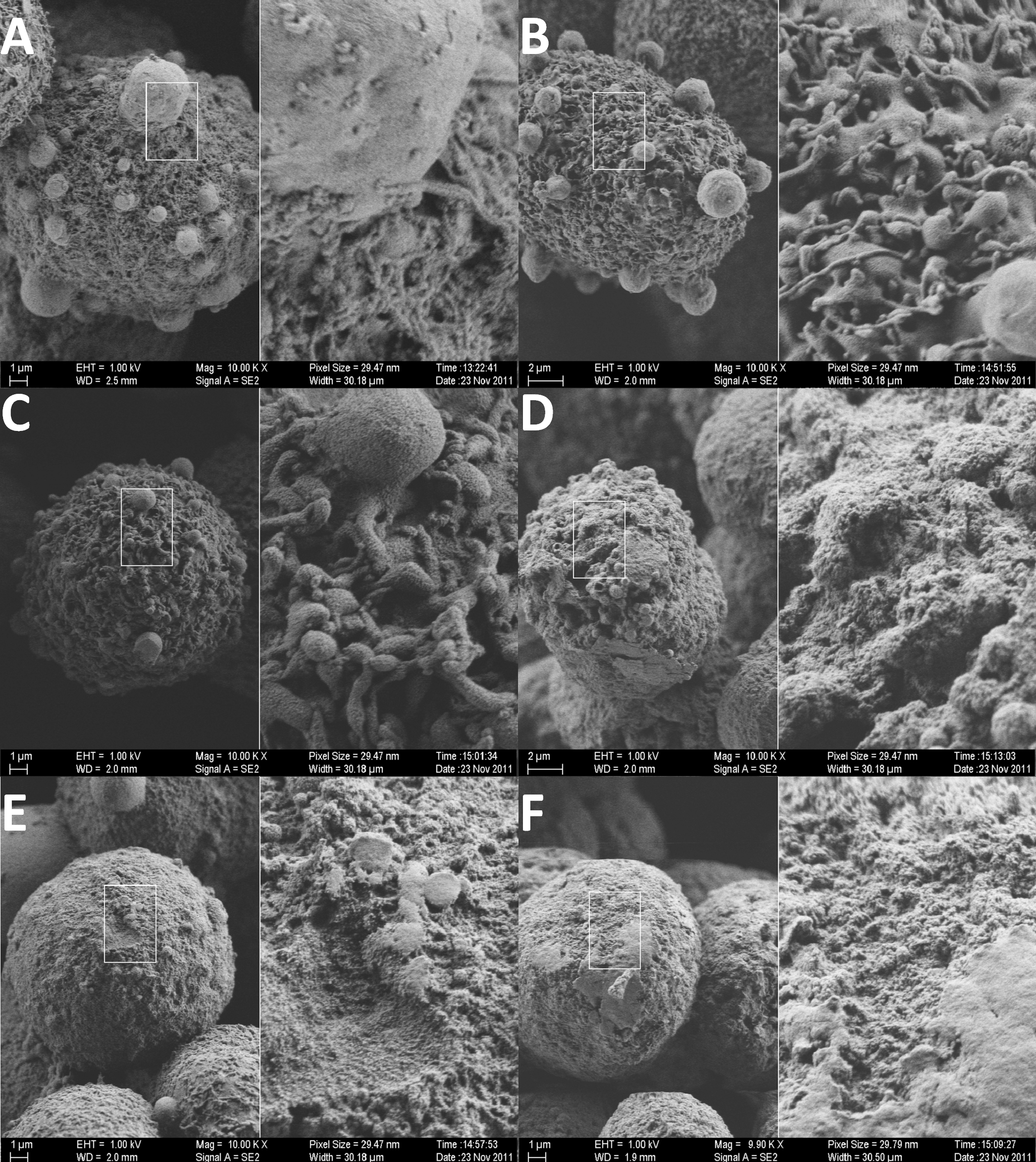

Supplement: Figure S6 — A) UW solution at -4oC B) HTS solution at -4oC C & E) UW solution at +4oC D & F) HTS solution at +4oC. In each image a second higher magnification section is displayed on the right hand side corresponding to the white outlined rectangle on the left. (TIF) [file pone.0069334.s006.tif]
